# Mitochondrial DNA m.3243A > G heteroplasmy affects multiple aging phenotypes and risk of mortality

**DOI:** 10.1038/s41598-018-30255-6

**Published:** 2018-08-08

**Authors:** Gregory J. Tranah, Shana M. Katzman, Kevin Lauterjung, Kristine Yaffe, Todd M. Manini, Stephen Kritchevsky, Anne B. Newman, Tamara B. Harris, Steven R. Cummings

**Affiliations:** 1 California Pacific Medical Center Research Institute, San Francisco, CA 94107 USA; 2 L.A. Eye Center and Clinic, Los Angeles, CA 90037 USA; 3 Departments of Psychiatry, Neurology, and Epidemiology, University of California, San Francisco and the San Francisco VA Medical Center, San Francisco, CA 94121 USA; 4 Department of Aging and Geriatric Research, University of Florida, Gainesville, FL 32601 USA; 5 Sticht Center on Aging, Wake Forest School of Medicine, Winston-Salem, NC 27157 USA; 6 Department of Epidemiology, University of Pittsburgh, Pittsburgh, PA 15213 USA; 7 Intramural Research Program, Laboratory of Epidemiology and Population Sciences, National Institute on Aging, Bethesda, MD 20892 USA

## Abstract

Mitochondria contain many copies of a circular DNA molecule (mtDNA), which has been observed as a mixture of normal and mutated states known as heteroplasmy. Elevated heteroplasmy at a single mtDNA site, m.3243A > G, leads to neurologic, sensory, movement, metabolic, and cardiopulmonary impairments. We measured leukocyte mtDNA m.3243A > G heteroplasmy in 789 elderly men and women from the bi-racial, population-based Health, Aging, and Body Composition Study to identify associations with age-related functioning and mortality. Mutation burden for the m.3243A > G ranged from 0–19% and elevated heteroplasmy was associated with reduced strength, cognitive, metabolic, and cardiovascular functioning. Risk of all-cause, dementia and stroke mortality was significantly elevated for participants in the highest tertiles of m.3243A > G heteroplasmy. These results indicate that the accumulation of a rare genetic disease mutation, m.3243A > G, manifests as several aging outcomes and that some diseases of aging may be attributed to the accumulation of mtDNA damage.

## Introduction

Within each mitochondrion there are thousands of maternally inherited circular mtDNA molecules coding for mitochondrial genes critical to oxidative phosphorylation (or OXPHOS). These populations of mtDNA molecules can contain either normal or mutated DNA in a mixture known as heteroplasmy^1^. Studies of elderly populations demonstrate an age-related increase in heteroplasmic load due to mtDNA mutations and rearrangements in post-mitotic tissues including: heart, central nervous system, brain and skeletal muscle^2–7^. An increased heteroplasmic load leads to reduced OXPHOS enzymatic activity and may be responsible for age-related functional decline^3,4^. The greatest impact of this bioenergetics defect has been shown in organs with high energy requirements such as skeletal muscle, retina, auditory neuroepithelia, brain and heart^5–9^.

Because mitochondrial function is cell-type specific, a single mtDNA mutation may lead to a variety of mitochondrial diseases, depending on the tissue in which the mutation is expressed^10^. Bioenergetic impairment of mitochondria through high heteroplasmic load (>80% burden of a pathogenic mtDNA mutation) may play a large role in disease initiation or progression^11,12^. We previously reported associations between elevated mtDNA heteroplasmy levels and reduced neurosensory and mobility function in older persons^13^. Average heteroplasmy levels among 20 candidate mutations detected in this earlier study of elderly participants ranged from 5–32%^13^.

Mitochondrial diseases resulting from mtDNA mutations often involve dysfunctions across multiple functional domains, including: neurologic, sensory, movement, metabolic, and cardiopulmonary outcomes^14^. Among the most thoroughly studied and best characterized of these pathogenic mutations is m.3243A > G, which causes several mitochondrial diseases and physiological dysfunctions, including: Mitochondrial Encephalopathy, Lactic Acidosis, Stroke-like episodes (MELAS), Mitochondrial Myopathy, Leigh syndrome, Chronic Progressive External Ophthalmoplegia (CPEO), Maternally Inherited Deafness and Diabetes (MIDD), hypertrophic cardiomyopathy (HCM), kidney dysfunction, migraine, bowel dysmotility, muscle stiffness, and diabetes^14^. Heterogeneity in the phenotypic expression related to m.3243A > G mutation ranges from mild to severe symptoms (e.g. mild deafness to stroke-like episodes)^15,16^.

To date, m.3243A > G heteroplasmy has not been examined for clinical associations in an aging population. In the current study we quantified m.3243A > G heteroplasmy in a community-based cohort of men and women over age 70 years old and tested associations with age-related functioning and mortality.

## Results

A total of 789 Health ABC participants of African and European ancestry yielded complete mtDNA sequences for analysis, analysis including 371 men and 418 women aged 74.1 (mean) ±2.9 (SD) years. Baseline values for measures of strength, movement, and cognitive, metabolic, and cardiovascular function are detailed in Table 1. Numbers for mortality events are also detailed in Table 1. Mean sequencing coverage for m.3243A > G was 961X with a lower limit of 82X. Heteroplasmy at m.3243A > G ranged from 0–19% with a mean (SD) of 5.55% (3.6) and a median of 5.11%. Heteroplasmy detected in this study is comparable to that from previous chip-^17^ and NGS-based^18^ platforms.

**Table 1:** Baseline characteristics among 789 sequenced Health ABC.

Variable	n (%)
Sex	
Male	371 (47)
Female	418 (53)
Race	
White	396 (50)
Black	393 (50)
Clinic Site	
Memphis	391 (50)
Pittsburgh	398 (50)
Variable	Mean (SD)
Baseline age, years	74.1 (2.9)
Modified Mini-Mental State Examination	91.1 (5.4)
Digit Symbol Substitution Test	33.8 (14.2)
400 m walk speed (m/s)	1.24 (0.21)
Grip Strength (kg)	29.3 (10.5)
Systolic blood pressure (mm Mercury)	136.5 (21.4)
Pulse wave velociy (cm/sec)	930.5 (421.9)
Resting heart rate (beats/min)	67.7 (10.7)
Fasting glucose (uIU/mL)	104 (30.8)
Fasting insulin (uIU/mL)	8.6 (8.36)
Mortality events (mean follow-up, 12.7 years)	n (%)
Total	481 (61%)
Cancer	121 (15%)
Cardiovascular	94 (12%)
Dementia	82 (10%)
Stroke	36 (5%)

Number of mortality events for a mean of 12.7 years of follow-up.

We identified statistically significant cross-sectional associations with one measurement from each of the subsets of 9 phenotypes examined (Table 2). The linear associations presented herein achieved nominal significance (p < 0.05): Digit Symbol Substitution Test (DSST) (p = 0.04), fasting insulin (p = 0.04), pulse wave velocity (PWV) (p = 0.008), and grip strength (p = 0.02). No statistically significant associations with other phenotypes examined in this study: 400 meter (m) walk (p = 0.94), Modified Mini-Mental State Examination (3MS) (p = 0.49), fasting glucose (p = 0.90), Systolic blood pressure (SBP) (p = 0.30), and resting heart rate (RHR) (p = 0.13).

**Table 2:** Phenotype values among tertiles of m.3243A > G heteroplasmy, mean and standard error (SE).

m.3243A > G heteroplasmy	0–4%	4–6%	6–19%	Linear p-value
	Mean (SE)	Mean (SE)	Mean (SE)	
Digit Symbol Substitution Test Score	35.3 (0.73)	33.1 (0.73)	33.1 (0.73)	0.04
Fasting insulin (uIU/mL)	7.65 (0.55)	8.30 (0.55)	9.80 (0.55)	0.04
Pulse wave velociy (cm/sec)	890 (27.8)	928 (28.3)	976 (28.6)	0.008
Grip Strength (kg)	31.8 (0.43)	30.9 (0.43)	30.4 (0.43)	0.02
Modified Mini-Mental State Examination Score	91.5 (0.3)	90.8 (0.3)	90.9 (0.3)	0.49
400 m walk speed (m/s)	1.24 (0.013)	1.25 (0.013)	1.23 (0.013)	0.94
Systolic blood pressure (mm Mercury)	137.2 (1.3)	136 (1.3)	136.2 (1.3)	0.30
Resting heart rate (beats/min)	68.2 (0.67)	67.5 (0.67)	67.3 (0.66)	0.13
Fasting glucose (uIU/mL)	101.7 (1.89)	105.5 (1.9)	104.7 (1.9)	0.90

A total of 263 participants were included in each tertile of m.3243A > G heteroplasmy. All values were adjusted for age, sex, race, and clinic site.

**Figure 1:**
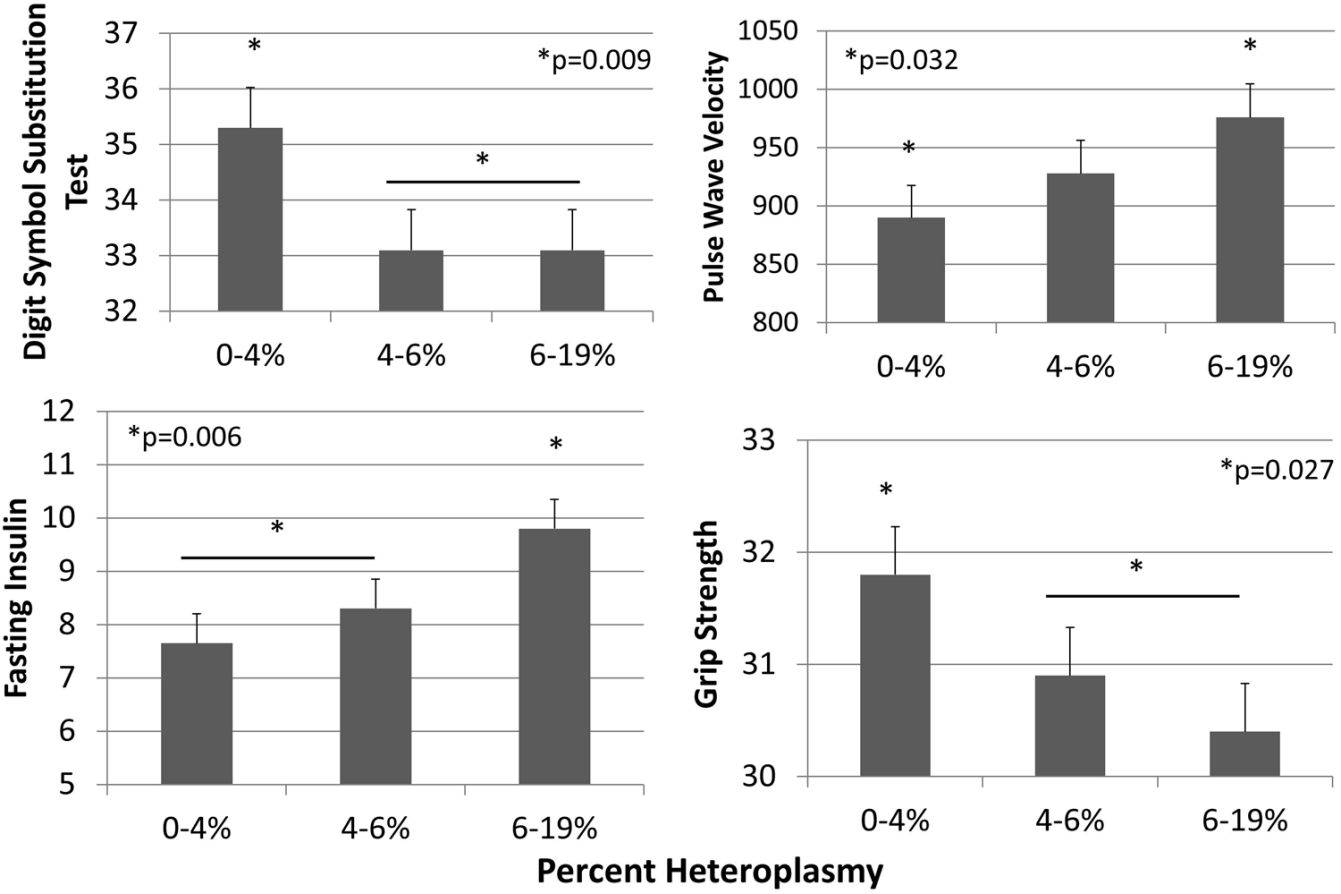
Mitochondrial m.3243A > G associations with digit symbol substitution test score (linear regression p = 0.04), fasting insulin (linear regression p = 0.04), pulse wave velocity (PWV, linear regression p = 0.008), and grip strength (linear regression p = 0.018) compared across tertiles of m.3243A > G heteroplasmy. A total of 263 participants were included in each tertile of m.3243A > G heteroplasmy. Bars indicate tertiles that were combined for analyses comparing phenotypes among low, middle, and high heteroplasmy levels. Asterisks (*) indicate statistically significant differences among individual and/or combined tertiles with p-values reported. Values and analyses adjusted for age, sex, race, and clinic site.

We further examined tertiles of m.3243A > G heteroplasmy for associations with DSST score, fasting insulin levels, PWV, and grip strength, (Table 2 and Fig. 1). A total of 263 participants were in each tertile of m.3243A > G heteroplasmy (%) with the following values: low (0.0–≤4%), middle (>4–≤6%), and high (>6–≤19%). DSST score (p = 0.009) was significantly higher for participants in the lowest tertile when compared with those in the middle and high tertiles. Fasting insulin levels were significantly lower (p = 0.006) for participants in the lowest tertile when compared with those in the middle and highest tertiles. PWV was significantly lower (p = 0.032) for participants in the lowest tertile when compared with those in the highest tertile. Grip strength (p = 0.027) was significantly higher for participants in the lowest tertile when compared with those in the middle and high tertiles.

**Figure 2:**
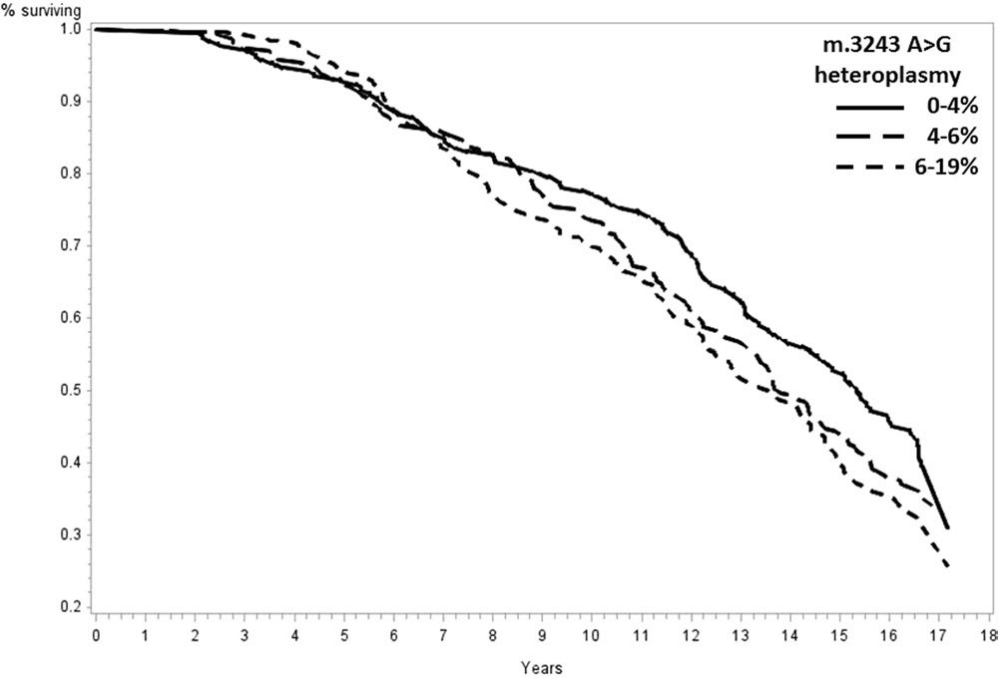
Mitochondrial m.3243A > G association with all-cause mortality. Survival was compared across tertiles of m.3243A > G heteroplasmy with a total of 263 participants included in each tertile. A significantly increased risk of all-cause mortality was observed for participants in the highest tertile of heteroplasmy when compared with those in the lowest tertile (Hazard Ratio [HR] = 1.25, 95% confidence interval [CI] = 1.01–1.56, p = 0.046). Analyses adjusted for age, sex, race, and clinic site.

**Figure 3:**
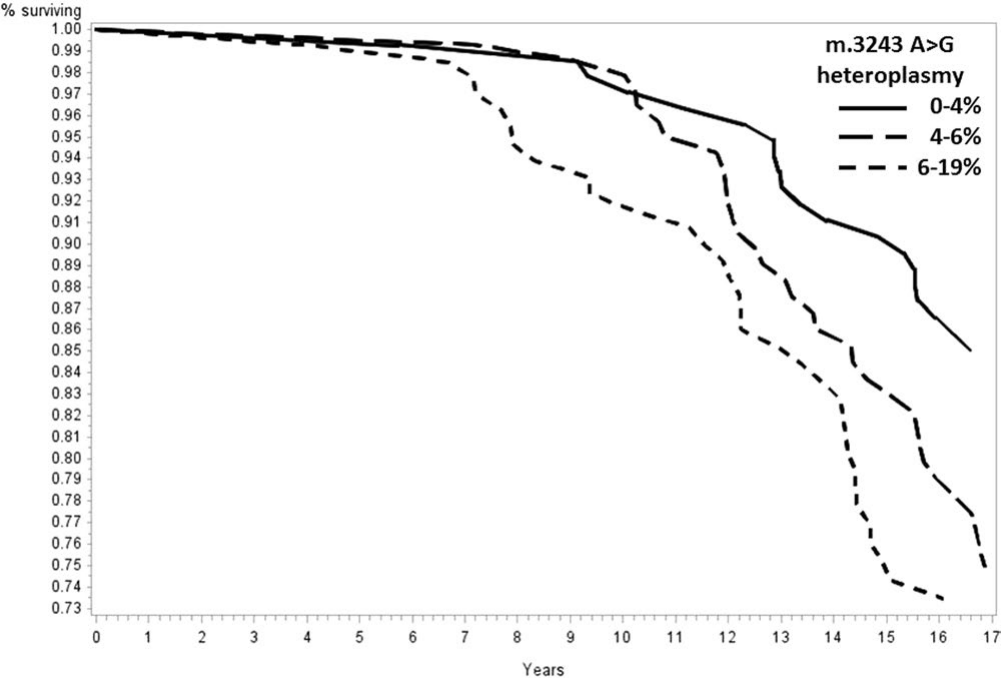
Mitochondrial m.3243A > G association with dementia-related mortality. Survival was compared across tertiles of m.3243A > G heteroplasmy with a total of 263 participants included in each tertile. A significantly increased risk of dementia mortality was observed for participants in the highest tertile of heteroplasmy when compared with those in the lowest tertile (HR = 1.96, 95% CI = 1.11–3.44, p = 0.02). Analyses adjusted for age, sex, race, and clinic site.

**Figure 4:**
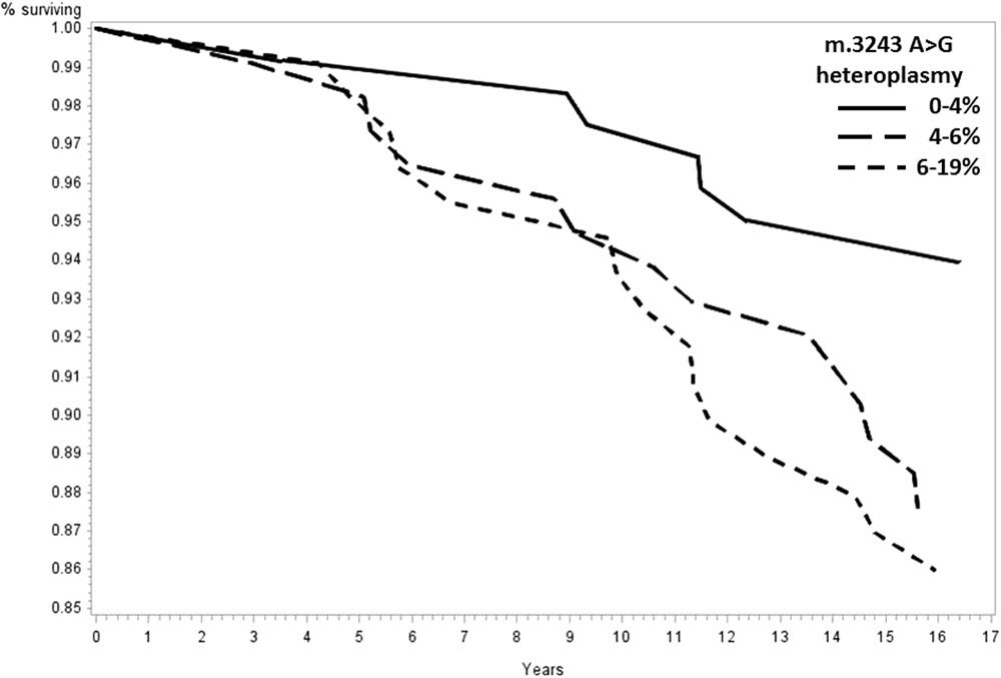
Mitochondrial m.3243A > G association with stroke-related mortality. Survival was compared across tertiles of m.3243A > G heteroplasmy with a total of 263 participants included in each tertile. A significantly increased risk stroke mortality was observed for participants in the highest tertile of heteroplasmy when compared with those in the lowest tertile (HR = 2.43, 95% CI = 1.00–5.97, p = 0.05). Analyses adjusted for age, sex, race, and clinic site.

Significantly increased risks of mortality were observed for participants in the highest tertile of heteroplasmy when compared with those in the lowest tertile for all-cause (Hazard Ratio [HR] = 1.25, 95% confidence interval [CI] = 1.01–1.56, p = 0.046), dementia (HR = 1.96, 95% CI = 1.11–3.44, p = 0.02), and stroke (HR = 2.43, 95% CI = 1.00–5.97, p = 0.05) mortality (Table 3). Kaplan-Meier curves demonstrate the cumulative incidence of all-cause (Fig. 2), dementia (Fig. 3), and stroke (Fig. 4) mortality. No statistically significant associations with cardiovascular (p = 0.32) and cancer (p = 0.16) mortality were identified (Supplementary Figs 1, 2).

**Table 3:** Cox proportional hazards models estimating hazard ratios and 95% confidence intervals among tertiles of m.3243A > G heteroplasmy for all-cause and cause-specific mortality.

	0–4%	m.3243A > G heteroplasmy	6–19%
		4–6%	
All-cause mortality, HR (95% CI)	1.00	1.18 (0.94–1.47, p = 0.16)	1.25 (1.01–1.56, p = 0.046)
Dementia, HR (95% CI)	1.00	1.58 (0.89–2.82, p = 0.12)	1.96 (1.11–3.44, p = 0.02)
Stroke, HR (95% CI)	1.00	2.11 (0.85–5.26, p = 0.11)	2.43 (1.00–5.97, p = 0.05)
Cancer, HR (95% CI)	1.00	1.40 (0.88–2.23, p = 0.17)	1.49 (0.96–2.32, p = 0.08)
CVD, HR (95% CI)	1.00	1.30 (0.78–2.15, p = 0.32)	1.28 (0.77–2.13, p = 0.35)

A total of 263 participants were included in each tertile of m.3243A > G heteroplasmy. All analyses were adjusted for age, sex, race, and clinic site.

## Discussion

Here we present novel findings on the role of sub-clinical levels of m.3243A > G heteroplasmy in an elderly population. Elevated m.3243A > G levels were associated with significantly impaired strength, cognition, cardiovascular, metabolic function, and mortality consistent with the diverse mitochondrial diseases and phenotypes typically associated with the m.3243A > G heteroplasmic load^14–16,19,20^. In the present study of non-disease presenting participants, circulating m.3243A > G heteroplasmy ranged from 0–19% with clear impairment at the highest tertiles.

In the context of mitochondrial diseases where the clinically relevant m.3243A > G mutation burden can reach levels over 90%, disease severity typically increases with elevated mutation burden^21,22^. Previous research has shown that m.3243A > G heteroplasmy levels of 10–30% are associated with type I and type II diabetes^23^ and autism^24^; heteroplasmy levels of 50–90% are associated with encephalomyopathies including MELAS^25^; and heteroplasmy levels of 90–100% lead to Leigh Syndrome or perinatal lethality^21^. In a large MELAS pedigree, 26 out of 27 living family members were m.3243A > G mutation positive^26^. Eighteen symptomatic m.3243A > G mutation carriers were identified without traditionally recognized stroke-like episodes but were diagnosed with diabetes, nephropathy, mild myopathy, cardiomyopathy, sensorineural hearing loss, cerebellar disease, and mental retardation. By contrast, eight m.3243A > G mutation carriers in this pedigree were asymptomatic demonstrating incomplete penetrance sometimes observed among mutation carriers. The clinical spectrum of heteroplasmic diseases has also been examined among twins both concordant and discordant for levels of mutation burden. Among monozygotic male twins carrying near-identical heteroplasmy levels of the pathogenic m.14487 T > C in different tissues, both were diagnosed with ptosis, optic atrophy, and myoclonic epilepsy^27^. By contrast, among monozygotic twins harboring a single large-scale mtDNA deletion, one brother harbored a high level of deleted mtDNA in muscle and exhibited ptosis, progressive external ophthalmoplegia, and proximal weakness while his twin brother had scarcely detectable deleted mtDNA molecules and was asymptomatic^28^. Among a set of dizygotic twins carrying the m.8344A > G mutation, one twin carried a high mutation load and was diagnosed with typical myoclonic epilepsy with ragged-red fibers (MERRF) while the other twin showed minimal mutation burden and was asymptomatic^29^.

Both *in vitro* and *in vivo* experiments have identified the molecular, structural, biochemical, and physiological impacts of m.3243A > G across the mutational spectrum and in multiple tissues. The m.3243A > G mutation located in the *tRNA*^*leu*^ gene produces quantitative differences in processing and steady state amounts of the *tRNA*^*leu*^ leading to amino acid misincorporation and impaired mitochondrial protein synthesis and assembly resulting in electron transport chain deficiency^30–32^. Biochemical studies of muscle in MELAS have also shown functional impairments of mtDNA-encoded complexes I, III, and IV^33–36^. In addition, widespread cellular dysfunction observed with elevated m.3243A > G mutation burden in MELAS impacts skeletal muscle, cerebral vasculature, and neuronal, endothelial, smooth muscle cells^33–38^. The consequences of the m.3243A > G mutation have been extensively examined using cytoplasmic hybrid (cybrid) models containing mtDNA from different sources placed in a uniform nuclear DNA background^39^ but have largely focused on on mtDNA sourced from clinically symptomatic MELAS patients carrying high m.3243A > G loads, > 90%^21,30^. However, MELAS patients often exhibit lower, tissue-specific m.3243A > G mutation loads^40,41^ and cybrids harboring ~20% m.3243A > G mutation loads exhibit reductions in cell surface area and elevated mtDNA density when compared with cybrids carrying only wild-type mtDNAs^21^. Respiratory chain dysfunction has also been reported in skeletal muscle^42^ and brain^43^ samples carrying m.3243A > G mutation levels under 10%.

A low-level of mtDNA heteroplasmy is commonly found in human populations^1,13,17,44,45^. Despite protective maternal mechanisms intended to minimize the transmission of mutated mtDNA^46,47^, heteroplasmy has been measured both in young children and during early adulthood. It remains uncertain whether the presence of heteroplasmy early in life is due to maternally transmitted mtDNA mutations^1^ or mutation acquisition during development^44,48,49^. Within the same individual, specific tissues may vary considerably with respect to their heteroplasmy and mutation loads^45,50–59^. Mutation load may accrue in post-mitotic tissues until a tissue-dependent threshold in the proportion of normal to mutated mtDNA is exceeded and the cells become bioenergetically deficient^60^. In general, mutation levels detected in blood are significantly lower than in other affected tissues including muscle, brain, liver, buccal mucosa, hair follicles, and urinary epithelium^26,27,45,57,59,61^. For example, similar levels of heteroplasmy were previously identified in two brain regions from the same individual while no heteroplasmy was detected in the blood^59^. Large studies examining mitochondrial genome-wide heteroplasmy across multiple tissues have demonstrated that heteroplasmy is extensive in nonsymptomatic subjects. For example, Naue *et al*.^45^ identified significant heteroplasmy in 88 out of 100 participants and detected the highest mutation levels in muscle and liver (69–79%), followed by brain, hair, and heart (30–37%), with the lowest levels in bone, blood, lung, and buccal cells (16–20%). Another study of 152 individuals examined heteroplasmy across twelve tissues obtained at autopsy and demonstrated that mutation burden at ten different mtDNA sites varied by tissue suggesting that both mutation- and tissue-driven processes have a role in driving mutation burden^61^. With regard to the m.3243A > G mutation, a study examining 61 individuals from 22 mutation-positive families demonstrated that mutation loads varied widely among five tissues^57^. Overall, urinary sediment exhibited the highest mutation burden followed by fibroblasts, cheek mucosa and hair roots, with blood showing the lowest proportion of mutant genomes^57^. The relationship between mtDNA mutation load and clinical phenotypes in humans has been a persistent subject for clinical diagnostics and recent studies have shown a superiority of urine epithelial cells over blood and muscle as a preferred non-invasive tissue for mtDNA mutation analysis^50–53,57^. The mechanisms responsible for inconsistent loading of mitochondrial heteroplasmy among specific cells^44,48,49^ and tissues is unknown; possibly genetic drift^62^ or selection against a particular mutation^63,64^ play significant roles. A better understanding of the mechanisms driving the expansion of mtDNA mutations and increased heteroplasmy load will further the development of interventions targeted to improved mitochondrial health^65–71^.

The current study has a number of strengths including the use of NGS sequencing and a platform designed specifically for complete mtDNA sequencing, and a large, community-based, well-characterized, biracial, longitudinal, cohort. Additionally, we were able to test our specific hypothesis that increased mtDNA heteroplasmy at the m.3234 > G mutation would be associated with impaired function across multiple phenotypes consistent with known mitochondrial disease impairments previously associated with this single mutation.

Although the rate of mitochondrial heteroplasmy accumulation is unknown in this population, we associated the measurements taken in the clinic with the mtDNA collected on a same-day visit thus ensuring that mortality was prospective and that the associations reported here are cross-sectional. Although the observed effect sizes for individual clinical measures associated with heteroplasmy are only moderately clinically significant, the ability to identify predictors of functional decline is critical to refining the associations between future disease onset and these clinical measures (e.g. of strength with disability or DSST with dementia). A limitation to this study is its absence of independent replication; the lack of associations for a number of phenotypes may be attributed to a limitation of sample size and tissue types (e.g. phenotypic examination may not have included all relevant tissues for mutation analysis). In order to confirm these findings, further population-level research including appropriate phenotypes, biospecimens, and design is necessary.

The Health ABC Study cohort is well-characterized and specifically designed for studies of aging-related impairments. Participants were generally healthy at the start of the study and it is likely that results from a single population may not be applied to all possible populations. These results indicate that the accumulation of a rare genetic disease mutation manifests as several aging outcomes and that some diseases of aging may be attributed to the accumulation of mtDNA damage. With further validation, measures of circulating mtDNA heteroplasmy may prove to be a valuable biomarker for identifying at-risk individuals who may benefit from early mitochondrial health interventions as well as for monitoring patients receiving mitochondrial therapies.

## Methods

### Participants

The Health, Aging, and Body Composition (Health ABC) Study is a prospective cohort of 3,075 community-dwelling men and women aged 70–79 years at recruitment in 1996–1997 and living in Memphis, TN, or Pittsburgh, PA. Participants were recruited within designated zip code areas from a random sample of white and black Medicare-eligible individuals. Participants had to be free of life-threatening cancer diagnoses and report no difficulty with the following activities of daily living: climbing 10 steps without resting or walking a quarter of a mile. Of the 3,075 participants, 51% were female and 41% were black. All participants signed written informed consents approved by the institutional review boards at the clinical sites (University of Tennessee Health Science Center, Memphis and University of Pittsburgh) and the coordinating center (University of California, San Francisco). All research and experimental protocols including participant recruitment, in-clinic assessments, blood collection and metabolic assays, and DNA sequencing were performed in accordance with relevant guidelines and regulations of the National Institute of Aging, the Health ABC Executive and Steering Committees, and the institutional review boards at the clinical sites (University of Tennessee Health Science Center, Memphis and University of Pittsburgh) and the coordinating center (University of California, San Francisco). All research and experimental protocols were also approved by the National Institute of Aging, the Health ABC Executive and Steering Committees, and the institutional review boards at the clinical sites (University of Tennessee Health Science Center, Memphis and University of Pittsburgh) and the coordinating center (University of California, San Francisco).

### Mitochondrial DNA sequencing

A total of 794 Health ABC participants were identified for mtDNA sequencing. Genomic DNA was extracted from buffy coat collected using PUREGENE® DNA Purification Kit from samples collected at the baseline visit (1997–1998). The entire mtDNA was sequenced using the Ovation® Human Mitochondrion Target Enrichment System (NuGEN, San Carlos, CA) on the Illumina MiSeq NGS platform. Briefly, DNA samples are first fragmented and end-repaired.

Barcoded sequencing adaptors are next ligated to the 5′ ends of the fragmented DNA and samples are combined for probe annealing and extension. Target enrichment prior to sequencing is accomplished with probes designed to independently target each strand of the mtDNA resulting in an enriched mtDNA library. After NGS sequencing, the FASTQ files were aligned with BWA^72^ to the Revised Cambridge Reference Sequence (rCRS, NC_012920.1). From the resulting BAM-files^73^, the bases for position 3243 relative to the rCRS were extracted and only bases with a PHRED-score ≥ 30 and a mapping quality ≥30 were used for the heteroplasmy detection. Heteroplasmy was derived for each m.3243A > G allele by counting the number of reads for each of the ‘G’ minor allele (MA) and ‘A’ reference allele (RA) and calculated as MA/(MA + RA).

### Strength and Mobility

Grip strength measured by handheld Jamar dynamometer^74^ and a timed walk of 400 meter (m) were assessed at the first clinical visit.

### Cognitive Function Testing

Two cognitive function tests were assessed among participants at the baseline clinical visit: Digit Symbol Substitution Test (DSST) and Modified Mini-Mental State Examination (3MS). DSST measures executive cognitive function^75,76^ and is calculated as the total number of items correctly coded in 90 seconds. 3MS is a general cognitive battery^77^ with possible scores ranging from 0 to 100. Higher DSST and 3MS scores indicate better cognitive functioning.

### Metabolic Measures

Fasting insulin and glucose were measured at the first clinical visit. Fasting insulin (uIU/mL) was measured via Microparticle Enzyme Immunoassay; Abbott Laboratories Diagnostics Division, South Pasadena, CA. Fasting glucose (mg/dL) was measured using Vitros Glucose; Johnson & Johnson; Rochester, NY USA.

### Cardiovascular Measures

Systolic blood pressure (SBP), resting heart rate (RHR), and pulse wave velocity (PWV) were measured at the first clinical visit. Sitting SBP was computed as the average of 2 measurements in millimeters of mercury. RHR (beats/minute) was automatically measured using a 12‐lead electrocardiogram. PWV (cm/s), a measure of arterial stiffness, was assessed transcutaneous Doppler flow probes; Parks Medical Electronics, Aloha, OR.

### Statistical analyses

Associations between m.3243A > G heteroplasmy and cognitive, movement and strength, cardiovascular, and metabolic function were examined using linear regression. Nominally significant linear associations (p < 0.05) among continuous predictor and outcome variables were further compared among tertiles of m.3243A > G heteroplasmy using ANOVA and general linear models were used to test differences among tertiles of heteroplasmy.

Vital status and cause of death were confirmed according to death certificates and hospital discharge summaries (when available) over an average of 12.7 years of follow-up. Cox proportional hazards models were used to estimate hazard ratios (HRs) and 95% confidence intervals [CIs] among tertiles of heteroplasmy for all-cause and cause-specific mortality. Kaplan-Meier curves were used to assess the cumulative incidence of all-cause and cause-specific mortality. All association and survival analyses were adjusted for age, sex, race, and clinic site using SAS version 9.4 (SAS Institute Inc, Cary, NC).

## Electronic supplementary material

Supplementary Figures
