# Supplementary material for: Mitochondrial DNA m.3243A > G heteroplasmy affects multiple aging phenotypes and risk of mortality
Source: Sci Rep. 2018 Aug 8;8:11887. doi: 10.1038/s41598-018-30255-6 (PMC6082898; doi:10.1038/s41598-018-30255-6)

Mitochondrial DNA m.3243A>G heteroplasmy affects multiple aging phenotypes and risk of mortality

Gregory J. Tranah [1*], Shana M. Katzman [2], Kevin Lauterjung [1], Kristine Yaffe [3], Todd M. Manini [4], Stephen Kritchevsky [5], Anne B. Newman [6], Tamara B. Harris [7], and Steven R. Cummings [1].

[1] California Pacific Medical Center Research Institute, San Francisco, CA, 94107, USA
[2] L.A. Eye Center and Clinic, Los Angeles, CA, 90037, USA
[3] Departments of Psychiatry, Neurology, and Epidemiology, University of California, San Francisco and the San Francisco VA Medical Center, San Francisco, CA 94121, USA
[4] Department of Aging and Geriatric Research, University of Florida, Gainesville, FL 32601, USA
[5] Sticht Center on Aging, Wake Forest School of Medicine, Winston-Salem, NC, 27157, USA
[6] Department of Epidemiology, University of Pittsburgh, Pittsburgh, PA, 15213, USA
[7] Intramural Research Program, Laboratory of Epidemiology and Population Sciences, National Institute on Aging, Bethesda, MD, 20892, USA

Supplementary Figure 1. Mitochondrial m.3243A>G not associated association with cancer-related mortality. Survival was compared across tertiles of m.3243A>G heteroplasmy with a total of 263 participants included in each tertile. No significantly increased risks with cancer-related mortality were observed Analyses adjusted for age, sex, race, and clinic site.

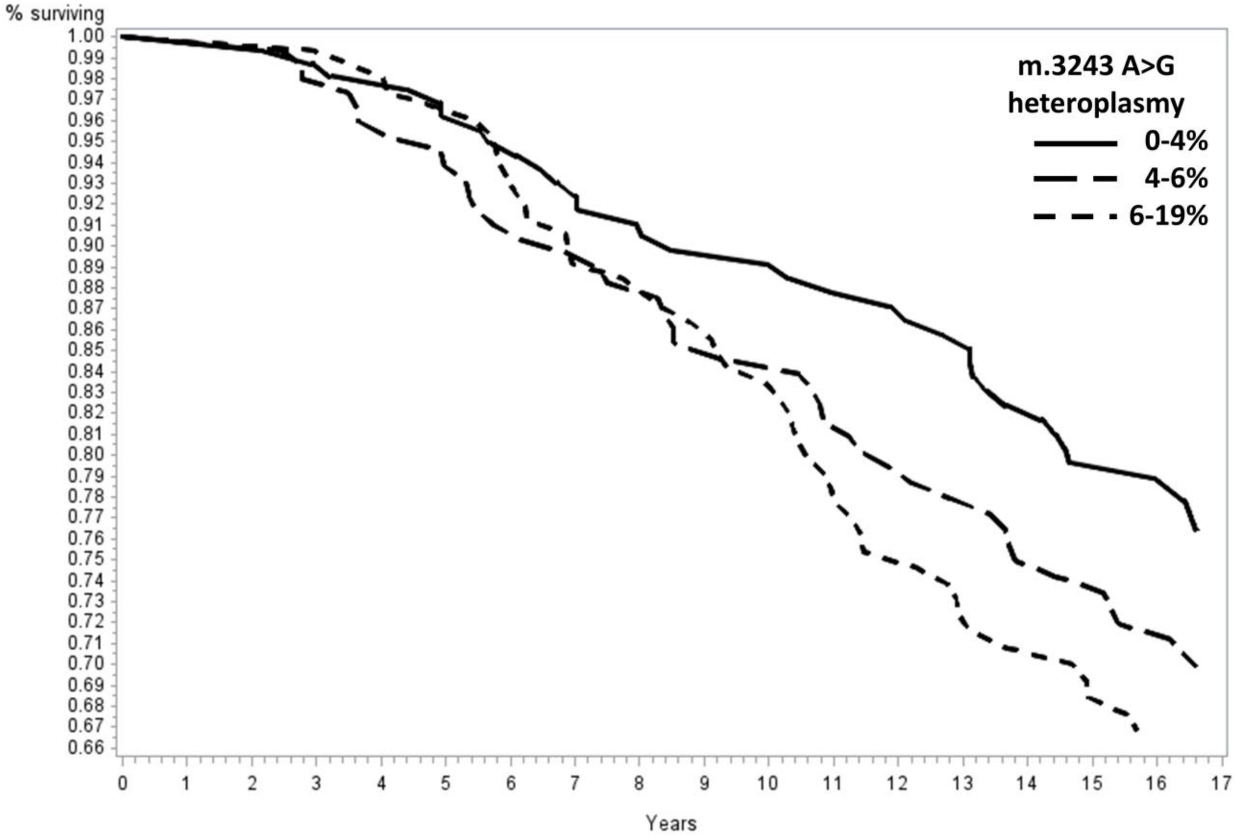

Supplementary Figure 2. Mitochondrial m.3243A>G association with CVD-related mortality. Survival was compared across tertiles of m.3243A>G heteroplasmy with a total of 263 participants included in each tertile. No significantly increased risks with CVD-related mortality were observed. Analyses adjusted for age, sex, race, and clinic site.

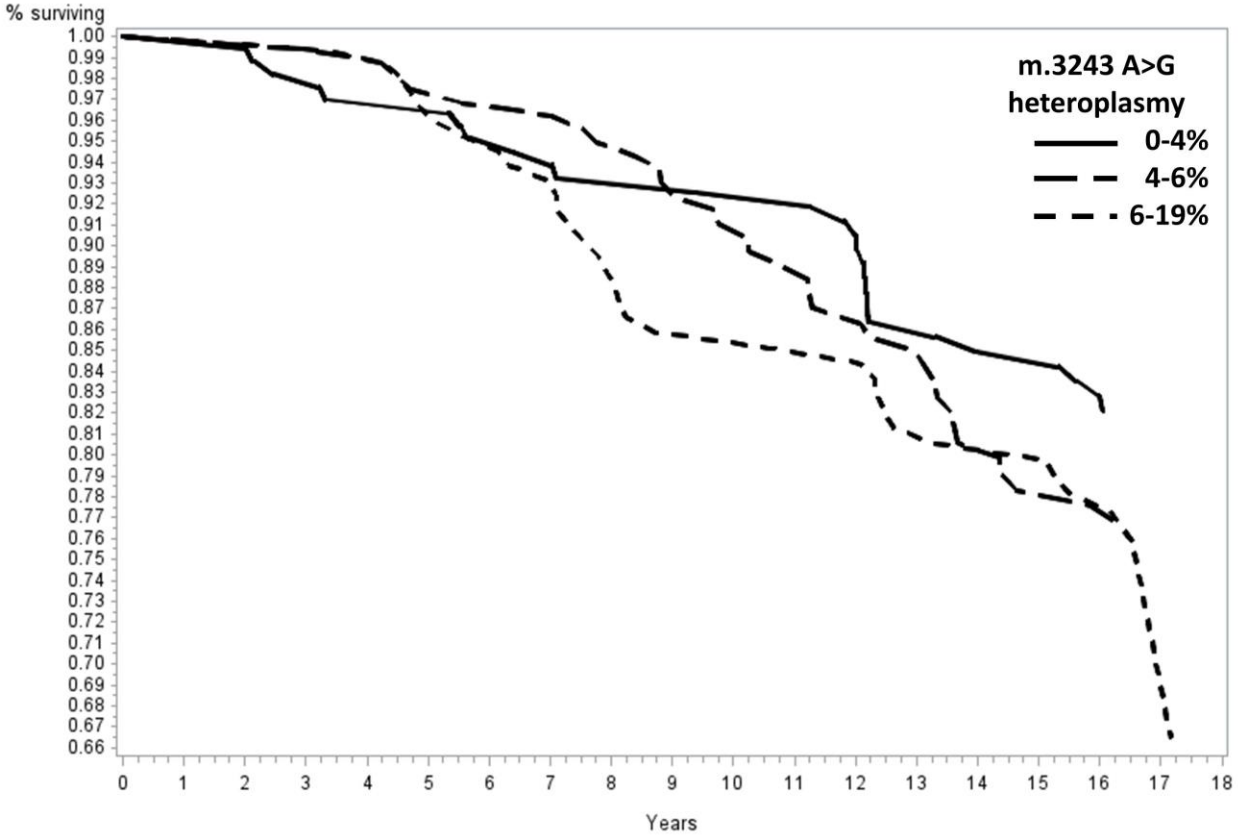

Supplement: Supplementary file 1 — Supplementary Figures [file 41598_2018_30255_MOESM1_ESM.pdf]
